# Prevalence and impact of Eustachian valve on the diagnosis of patent foramen ovale in patients ventilated for an acute respiratory distress syndrome

**DOI:** 10.1186/s13054-023-04670-9

**Published:** 2023-10-10

**Authors:** Florence Sanchez, Marine Goudelin, Bruno Evrard, Philippe Vignon

**Affiliations:** 1 Medical-Surgical Intensive Care Unit, Dupuytren Teaching Hospital, Limoges, France; 2 Medical Intensive Care Unit, Brive Hospital, Brive-la-Gaillarde, France; 3 Inserm CIC 1435, Dupuytren Teaching Hospital, Limoges, France; 4 Réanimation Polyvalente, CHU Dupuytren, 2 Ave. Martin Luther King, 87000 Limoges, France

Elective increase of right atrial (RA) pressure may contribute to reopening a *patent foramen ovale* (PFO) by reversing normal interatrial pressure gradient [1]. Accordingly, PFO is identified in 15 to 19% of patients ventilated for an acute respiratory distress syndrome (ARDS) due to increased right ventricular afterload [1, 2]. PFO diagnosis relies on transesophageal echocardiography (TEE) coupled with a contrast study. Eustachian valve, a remnant of the right *sinus venosus* valve located at the junction between the inferior vena cava (IVC) and RA, may hamper the identification of a PFO. We sought to assess the prevalence of persistent Eustachian valve and its impact on PFO depiction in ventilated adult ARDS patients.

This ancillary study included the subset of patients hospitalized in our ICU who previously participated in the multicenter ARCOFOP study [2]. The selection of this cohort was based on the availability of digitally stored TEE loops for off-line analysis. We studied 79 ARDS patients [median age: 58 (25th–75th percentiles: 47–68) years; 59 men; SAPSII: 43 (32–53); SOFA: 7 (4–10); PaO_2_/FiO_2_: 95 (72–138)] under protective mechanical ventilation [tidal volume: 6.2 (5.8–7.0) ml/kg; PEEP: 10 (8–12) cmH_2_O; plateau pressure: 25 (24–27) cmH_2_O] for moderate-to-severe ARDS unrelated to SARS-CoV-2. Data analysis was restricted to the interpretation of TEE contrast studies (20 mL of agitated saline injected through the central venous catheter located in the superior vena cava) in the longitudinal view of the interatrial septum (bicaval view). RA opacification was deemed complete when microbubbles enhanced its entire surface and reached the *fossa ovalis* for a least three cardiac cycles. PFO was diagnosed when microbubbles entered the left atrium through the foramen ovale during the first three cardiac cycles following full RA opacification, and right-to-left interatrial shunt was assessed semiquantitatively using a previously proposed 3-grade scale [3]. The presence of Eustachian valve and its length was systematically assessed.

Eustachian valve was identified in 19 patients (24%) and its median length reached 15 [11.5–16.5] mm. PFO was identified in 19 patients (24%), with mild-to-moderate right-to-left shunt (grade 1: *n* = 13; grade 2: *n* = 6 patients; grade 3: *n* = 0). RA opacification was incomplete in 13 patients (16%), 12 of them having Eustachian valve. Incomplete RA opacification was more frequent in patients with Eustachian valve than in their counterparts (12/19 (63%) versus 1/60 (2%): *p* < 0.001), whereas PFO prevalence was similar (5/19 (26%) versus 14/60 (23%): *p* = 0.767). In 3 patients with incomplete RA opacification due to Eustachian valve, injection of microbubbles through the IVC (femoral vein) enabled complete RA opacification and PFO identification in 2 of them with a grade 2 interatrial shunt (Fig. 1).

**Fig. 1:**
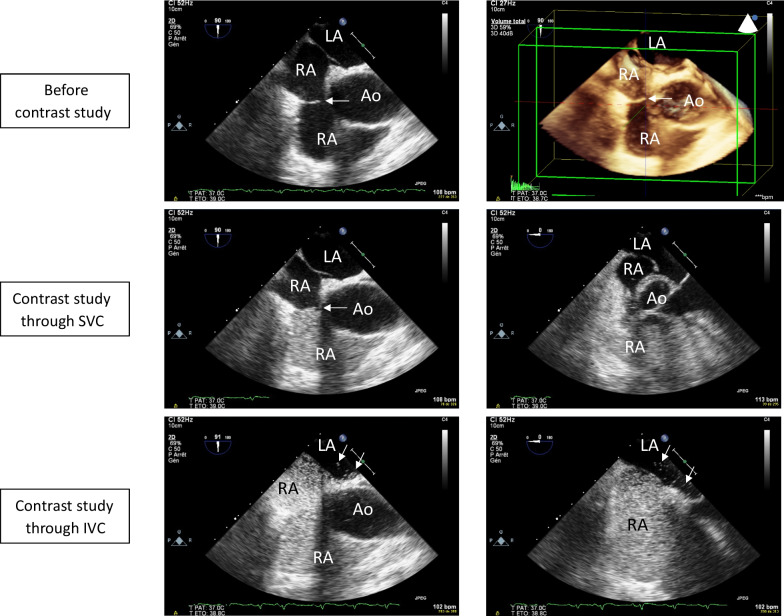
Transesophageal contrast study conducted in a ventilated ARDS patient with a prominent Eustachian valve and a *patent foramen ovale* with grade 2 interatrial right-to-left shunt. The bicaval view depicts an Eustachian valve in both two- and real-time three-dimensional imaging (upper panels, arrow). When the contrast study is performed through the superior vena cava (SVC), the microbubbles fail to reach the *fossa ovalis*, and the right atrium is incompletely opacified due to the presence of the Eustachian valve (arrow), in both the longitudinal bicaval view (90°, middle left panel) and transverse view (0°, middle right panel). When the contrast study is performed through the inferior vena cava (IVC), right atrial opacification is complete and allows the identification of a *patent foramen ovale* with microbubbles entering the left atrium during the first three cardiac cycle (arrows), in both the longitudinal bicaval view (90°, lower left panel) and transverse view (0°, lower right panel). Abbreviations: *LA* left atrium, *RA* right atrium, *Ao* ascending aorta, *SVC* superior vena cava, *IVC* inferior vena cava

Eustachian valve was identified in one-fourth of our patients under protective ventilation for a moderate-to-severe ARDS and was not associated with a higher prevalence of PFO. The 24% prevalence of PFO in our cohort was similar to that reported previously in patients with similar characteristics [1] and to the 27% incidence reported in a large necropsy study performed in the general population [4]. Actual prevalence of PFO may have been underestimated since RA opacification was incomplete in 16% of our patients and may have led to false-negative results of contrast studies. During fetal development, the Eustachian valve directs oxygenated blood flow from the IVC toward the foramen ovale and the systemic circulation through the left atrium. Usually, it involutes during the first years of life. Its persistence in adulthood can prevent spontaneous closing of PFO by directing blood from the IVC directly toward the interatrial septum. Consequently, it can lead to false-negative results of TEE contrast study in precluding the microbubbles injected through the superior vena cava to reach the interatrial septum, as in 12/13 of our patients with incomplete RA opacification. Although Gin et al. [5] recommended injecting the microbubbles through the femoral vein to increase the sensitivity of echocardiography contrast study for the detection of PFO, they did not report on the presence of Eustachian valve. Interestingly, 2 of our 3 patients with a negative TEE contrast study who underwent an additional injection of microbubbles through the IVC (femoral vein) exhibited a PFO with a grade 2 interatrial shunt, which was not initially identified. In contrast, the presence of a negative conventional TEE contrast study with full RA opacification allows to confidently rule out PFO [3]. The present study is limited by its retrospective design and by the lack of systematic injection of microbubbles through the IVC in patients with incomplete RA opacification, which precluded determining the actual proportion of false-negative TEE contrast study.

Eustachian valve was present in one-fourth of our patients ventilated for a moderate-to-severe ARDS unrelated to SARS-CoV-2 and was closely associated with incomplete RA opacification during TEE contrast study. In patients with Eustachian valve and incomplete RA opacification, an additional injection of microbubbles through the IVC should be performed to avoid false-negative results.

## Data Availability

The datasets used and/or analyzed during the current study are available from the corresponding author on reasonable request.

## Abbreviations

RA: Right atrium
ARDS: Acute respiratory distress syndrome
PFO: Patent foramen ovale
TEE: Transesophageal echocardiography
IVC: Inferior vena cava

## References

[1] Mekontso Dessap A, Boissier F, Leon R, Carreira S, Campo FR, Lemaire F, Brochard L (2010). Prevalence and prognosis of shunting across patent foramen ovale during acute respiratory distress syndrome. Crit Care Med.

[2] Lhéritier G, Legras A, Caille A, Lherm T, Mathonnet A, Frat JP, Courte A, Martin-Lefèvre L, Gouëllo JP, Amiel JB, Garot D, Vignon P (2013). Prevalence and prognostic value of acute cor pulmonale and patent foramen ovale in ventilated patients with early acute respiratory distress syndrome: a multicenter study. Intensive Care Med.

[3] Schneider B, Zienkiewicz T, Jansen V, Hofmann T, Noltenius H, Meinertz T (1996). Diagnosis of patent foramen ovale by transesophageal echocardiography and correlation with autopsy findings. Am J Cardiol.

[4] Hagen PT, Scholz DG, Edwards WD (1984). Incidence and size of patent foramen ovale during the first 10 decades of life: an autopsy study of 965 normal hearts. Mayo Clin Proc.

[5] Gin KG, Huckell VF, Pollick C (1993). Femoral vein delivery of contrast medium enhances transthoracic echocardiographic detection of patent foramen ovale. J Am Coll Cardiol.

